# Clinical efficacy and safety of Shenqi Fuzheng injection for the treatment of chronic heart failure: Protocol for a meta-analysis and systematic review

**DOI:** 10.1097/MD.0000000000018556

**Published:** 2019-12-27

**Authors:** Chuan Wang, Dongfeng Yao, Pan Zhang, Xiaowei Xie, Bin Wang, Jiping Liu, Zhen Zhang

**Affiliations:** a Department of Pharmacology, College of Pharmacy, Shaanxi University of Chinese Medicine, Xianyang; b Key Laboratory of Pharmacodynamics and Material Basis of Chinese Medicine of Shaanxi Administration of Traditional Chinese Medicine; c Subject Innovation Team of Shaanxi University of Chinese Medicine; d Shaanxi Collaborative Innovation Center of Chinese Medicinal Resources Industrialization, Shaanxi University of Chinese Medicine, China.

**Keywords:** chronic heart failure, meta-analysis, protocol, Shenqi Fuzheng injection, systematic review

## Abstract

**Background:**

Chronic heart failure (CHF) is one of the most serious cardiovascular diseases. Shenqi Fuzheng injection (SQFZI), as a Chinese herbal injection, is usually used for the treatment of CHF. However, the clinical evidence of SQFZI for the treatment of CHF is unclear.

**Methods:**

Two researchers will dependently search literatures of SQFZI for CHF from Chinese National Knowledge Infrastructure database, VIP database, Chinese Biological and Medicine database, Wangfang database, MEDLINE, EMBASE, Cochrane Library and Web of Science. These inclusive data of included studies will be conducted by RevMan 5.3 software.

**Results:**

This meta-analysis and systematic review will provide a series of outcome measures to verify clinical efficacy and safety of SQFZI for treating CHF, including New York Heart Association (NYHA) function classification, left ventricular ejection fraction, left ventricular end-diastolic dimension, cardiac output, stroke volume, brain natriuretic peptide, N-terminal pro-brain natriuretic peptide, and adverse events.

**Conclusions:**

This meta-analysis and systematic review will provide up-to-date clinical evidence to assess SQFZI treatment efficacy for CHF patients.

## 1 Introduction

Chronic heart failure (CHF) is a heart disease which is caused by myocardial damage, heart overload and systolic dysfunction.^[1]^ The high incidence and mortality of CHF have brought enormous burden and challenge to social economy and medical resources.^[2]^ CHF is a progressive disease, even if no new myocardial damage is produced from the onset.^[3]^ Patients show no obvious clinical signs and symptoms, but this does not mean the disease will not further develop. Currently, neurohormonal antagonists (ACEI, β-blockers, angiotensin receptor blockers and mineralocorticoid receptor antagonists) are recommended as cornerstone treatments for CHF.[^4^,^5^] However, these are unsatisfactory in terms of clinical efficacy and safety.^[6]^ The Shenqi Fuzheng injection (SQFZI) is a Chinese herbal injection which is mainly composed of *Radix Codonopsis* (Dangshen) and *Radix Astragali* (Huangqi).^[7]^ SQFZI is approved by the China Food and Drug Administration (CFDA) and has already achieved positive results for the treatment of CHF in one clinical trial,^[8]^ although more evidence is needed for its efficacy to be approved in clinical guidelines.[^9^,^10^] Recently, there have been more randomized controlled trials (RCTs) testing SQFZI's efficacy for treating CHF. We therefore conducted a systematic evaluation of the clinical efficacy and safety of SQFZI combined with Western medicine for treating CHF.

## 2 Methods

### 2.1 Inclusion criteria

#### 2.1.1 Type of study

Studies will be included this meta-analysis, regardless of publication countries and language. These included studies of SQFZI for treating CHF.

#### 2.1.2 Type of patients

According to “Guidelines for the Diagnosis and Treatment of Chronic Heart Failure in China” or “American College of Cardiology/American Heart Association (ACC/AHA) guidelines 2016”,^[11]^ patients only contains CHF, other diseases such as diabetes, hypothyroidism, gallbladder disease are excluded.

#### 2.1.3 Type of intervention

For the treatment group, SQFZI will be intravenously instilled daily on the basis of conventional therapy with CHF patients, while in the control group, only conventional therapy for CHF treatment alone.

#### 2.1.4 Type of outcome measures

*Primary outcomes:*

- NYHA function classification;
- Clinical total effective rate.

*Secondary outcomes:*

- Left ventricular ejection fraction (LVEF);
- Left ventricular end-diastolic dimension (LVEDD);
- Cardiac output (CO);
- Stroke volume (SV);
- Brain natriuretic peptide (BNP);
- N-terminal pro-brain natriuretic peptide (NT-proBNP);
- Hypersensitive C-reactive protein (hs-CRP);
- Adverse events.

### 2.2 Search strategy for the included studies

#### 2.2.1 Database search

We will search for literatures on SQFZI treatment of CHF from the following 7 databases:

1. China National Knowledge Infrastructure (CNKI)
2. Wanfang Database
3. Chinese VIP Information (VIP)
4. Chinese Biomedical Literature Database (CBM)
5. MEDLINE
6. The Cochrane Library
7. Web of Science

#### 2.2.2 Searching other resources

Meanwhile, we will also search Chinese Clinical Trials Registry (ChiCTR) (http://www.chictr.org.cn/) to collect data of included RCTs with SQFZI for treating CHF.

#### 2.2.3 Search strategy in electronic database

These main keywords are: Shenqi Fuzheng Injection, CHF, randomized controlled trials. Details of search strategy in PubMed as follow:

#1 (“heart failure, chronic” [MeSH Terms]) OR (“heart failure∗” [Title/Abstract]) OR (“chronic heart failure∗” [Title/Abstract]) OR (“coronary heart disease∗” [Title/Abstract]) OR (“coronary artery disease” [Title/Abstract])
#2 (“Shenqi Fuzheng, Injection” [MeSH Terms]) OR (“Shenqi Fuzheng Injection∗” [Title/Abstract]) OR (“Shenqi Fuzheng Injectables∗” [Title/Abstract]) OR (“SQFZ injection∗” [Title/Abstract]) OR (“Dangshen and *Huangqi*, Injection” [Title/Abstract]) OR (“*Radix Codonopsis* and *Radix Astragali,* injection” [Title/Abstract])
#3 (“Randomized, controlled trial” [MeSH Terms]) OR (“Randomized controlled trial∗” [Title/Abstract]) (“clinical study” [Title/Abstract]) OR (“Clinical Trial” [Title/Abstract]) OR (“Controlled study∗” [Title/Abstract]) OR (“Controlled Trial∗” [Title/Abstract])
#1 AND #2 AND #3

### 2.3 Data collection and analysis

#### 2.3.1 Study selection

**Figure 1:**
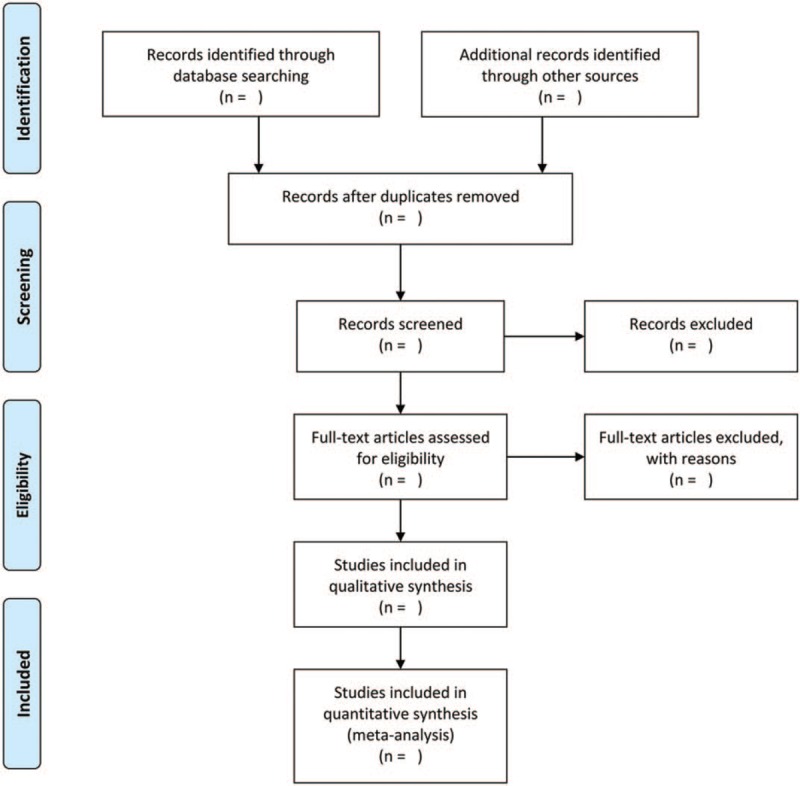
PRISMA flowchart of selection studies.

Through database searching, duplicate studies will be deleted and remaining eligible studies will be transferred to EndNote X9 software (Thomson Reuters, Inc, New York, NY). Two researchers will carefully scan the titles and abstracts of studies to exclude studies that is not related to the topic. Full-text studies will further screen studies that may meet the inclusion criteria. If necessary, the third researcher will discuss into disagreement of selection studies. Details of study selection will be shown as in a PRISMA flowchart (Fig. 1).

#### 2.3.2 Data extraction

Two researchers independently screened the literature, following the inclusion and exclusion criteria. When there were differences, consensus was reached through discussion. If necessary, a third researcher joined the discussion. Extracted data included basic characteristics, including first author, publication year, country, patients’ mean age, NYHA class, intervention methods, sample size, and outcomes.

#### 2.3.3 Heterogeneity analysis

The risk ratio (RR) was results of dichotomous variables with 95% confidence intervals (CI 95%). The mean difference (MD) was the results of the continuous variables with CI 95%. A heterogeneity test was used. If *P* > .1, the fixed effect model was used for meta-analysis. Otherwise, the random effect model was used.

### 2.4 Risk of bias

The Cochrane Handbook suggests seven considerations for evaluating risk of bias as follow: random sequence generation method, allocation concealment, blinding of participants and personnel, blinding of outcome assessment, incomplete outcome data, selective reporting, and other sources. Each consideration was divided into three levels for the selected studies: “low risk”, “high risk”, or “unclear”.

### 2.5 Publication bias

When more than ten RCTs are included, the symmetry of the funnel plot will be used to assess to evaluate whether the funnel plot is symmetrical. If necessary, we will also use STATA 12.0 software to evaluate stability of included studies.

### 2.6 Subgroup analysis

Heterogeneity is one of the main factors affecting the results of meta-analysis. So, subgroup analysis will be used to analyze the source of heterogeneity. We will perform a subgroup analysis from the following points to determine the source of heterogeneity:

- Different treatment courses
- Different daily doses
- People of different skin colors
- Inclusion of differences in studies quality

### 2.7 Sensitivity analysis

Sensitivity analysis is an important method used in meta-analysis to assess the robustness and reliability of results. The commonly used method is to eliminate each of the included studies one by one and then combine the effect quantities, change the inclusion of exclusion criteria or eliminate certain types of literature and then combine effect sizes.

## 3 Discussion

CHF is a global health problem.^[12]^ While medical technology has improved its treatment, disease incidence and mortality has increased year by year. Myocardial injury is the main pathogenesis of CHF, which can lead to the complex clinical syndrome of low ventricular pumping and filling function.^[13]^ In China, traditional Chinese medicine (TCM) has been widely used for treating heart failure.[^14^,^15^] SQFZI is a new type of TCM injection and is mainly composed of Radix Codonopsis (Dangshen) and Radix Astragali (Huangqi), 2 herbal medicines which have been used in China for thousands of years.^[16]^ Hence, we will conduct a meta-analysis to evaluate efficacy and safety of SQFZI for treating CHF. However, due to methodological quality, different doses, and only included English and Chinese database, it is necessary to carry out large sample, high-quality RCTs in the future to verify the treatment's therapeutic effect and provide more convincing evidence for its use in the treatment of CHF.

## Author contributions

**Conceptualization:** Chuan Wang.

**Formal analysis:** Pan Zhang, Dongfeng Yao, Xiaowei Xie.

**Data curation:** Chuan Wang, Jiping Liu, Bin Wang.

**Funding acquisition:** Chuan Wang.

**Writing - original draft:** Dongfeng Yao, Zhen Zhang.

**Writing - review & editing:** Dongfeng Yao, Chuan Wang.

**Software:** Dongfeng Yao.

Chuan Wang orcid: 0000-0002-8016-0113.
